# Combined Layer-by-Layer/Hydrothermal Synthesis of Fe_3_O_4_@MIL-100(Fe) for Ofloxacin Adsorption from Environmental Waters

**DOI:** 10.3390/nano11123275

**Published:** 2021-12-02

**Authors:** Michela Sturini, Constantin Puscalau, Giulia Guerra, Federica Maraschi, Giovanna Bruni, Francesco Monteforte, Antonella Profumo, Doretta Capsoni

**Affiliations:** 1 Department of Chemistry, University of Pavia, 27100 Pavia, Italy; michela.sturini@unipv.it (M.S.); giulia.guerra@itb.cnr.it (G.G.); federica.maraschi@unipv.it (F.M.); antonella.profumo@unipv.it (A.P.); 2 C.S.G.I. (Consorzio Interuniversitario per lo Sviluppo dei Sistemi a Grande Interfase) & Department of Chemistry, Physical Chemistry Section, University of Pavia, 27100 Pavia, Italy; constantin.puscalau@nottingham.ac.uk (C.P.); giovanna.bruni@unipv.it (G.B.); francesco.monteforte01@universitadipavia.it (F.M.); 3 The GlaxoSmithKline Neutral Laboratories for Sustainable Chemistry, University of Nottingham, Jubilee Campus, Nottingham NG7 2TU, UK; 4 Istituto di Tecnologie Biomediche, ITB-CNR, 20054 Segrate, Milano, Italy

**Keywords:** combined layer-by-layer/hydrothermal synthesis, fluoroquinolone antibiotic, iron-based metal-organic frameworks, adsorption, wastewater treatment, magnetic remediation, polluted waters

## Abstract

A simple not solvent and time consuming Fe_3_O_4_@MIL-100(Fe), synthesized in the presence of a small amount of magnetite (Fe_3_O_4_) nanoparticles (27.3 wt%), is here presented and discussed. Layer-by-layer alone (20 shell), and combined layer-by-layer (5 shell)/reflux or /hydrothermal synthetic procedures were compared. The last approach (Fe_3_O_4_@MIL-100_H sample) is suitable (i) to obtain rounded-shaped nanoparticles (200–400 nm diameter) of magnetite core and MIL-100(Fe) shell; (ii) to reduce the solvent and time consumption (the layer-by-layer procedure is applied only 5 times); (iii) to give the highest MIL-100(Fe) amount in the composite (72.7 vs. 18.5 wt% in the layer-by-layer alone); (iv) to obtain a high surface area of 3546 m^2^ g^−1^. The MIL-100(Fe) sample was also synthesized and both materials were tested for the absorption of Ofloxacin antibiotic (OFL). Langmuir model well describes OFL adsorption on Fe_3_O_4_@MIL-100_H, indicating an even higher adsorption capacity (218 ± 7 mg g^−1^) with respect to MIL-100 (123 ± 5 mg g^−1^). Chemisorption regulates the kinetic process on both the composite materials. Fe_3_O_4_@MIL-100_H performance was then verified for OFL removal at µg per liter in tap and river waters, and compared with MIL-100. Its relevant and higher adsorption efficiency and the magnetic behavior make it an excellent candidate for environmental depollution.

## 1. Introduction

Metal-organic frameworks (MOFs) are inorganic–organic hybrid compounds based on the coordinative bonding of metal ions and organic ligands, first investigated by Yaghi et al. [1]. They are crystalline porous materials, with extremely high surface areas, tunable pore size, cages and tunnels, and abundant functional sites that make them such outstanding adsorbent materials compared to the traditional ones. Due to their peculiar attractive properties, MOFs’ use has grown strongly in several fields, i.e., food [2], gas storage and separation [3,4,5,6], clinical [7] and biological applications [8], and recently in environmental [9] and analytical chemistry [10]. Despite superior performance for many applications, MOFs are often rather fragile and mechanically and thermally unstable compared to other competing materials. Their decomposition temperature and mechanical features vary in a rather wide range, depending on the choice of the metal center and the linkers, the structure, the synthesis process and parameters [11,12]. Undoubtedly the instability is a crucial point, and recent papers have been published to design and synthesize MOFs displaying chemical, mechanical and thermal stability [13,14].

More than 20,000 MOFs were proposed in the last decade, and, among them, the MIL (Materials of Institute Lavoisier) series have drawn wide attention for both their high surface areas and their stability in aqueous media. In particular, the MIL100(Fe) has been chosen because it is environmentally benign, chemically robust, thermally stable up to 270 °C, and displays surface areas in the 1000–4500 m^2^ g^−1^ range [15,16,17,18]. It is composed of Fe(III) metal ions and benzene-1,3,5-tri-carboxylate organic linker. It displays a cubic crystal structure [16] that contains mesoporous cages (25–29 Å) and microporous windows (5–9 Å), as reported in the literature [19] and deposited in the Cambridge Crystallographic Data Centre (CCDC 640536). It was used for gas adsorption and separation [20], catalytic applications [16,21], drug delivery [22,23], dyes, metal ions [24,25,26,27] and pesticides adsorption [28]. MIL100(Fe) has been recently employed also for pharmaceuticals removal from polluted waters, such as diclofenac [29], tetracycline [30,31] and levofloxacin [32]. 

To date, pharmaceuticals are considered contaminants of emerging concern (CEC) because of their widespread diffusion in surface waters, even at trace levels (ng L^−1^), their recalcitrant behavior in the wastewater treatment plants, and their recognized adverse effects on aquatic organisms. It is well recognized that urban wastewater treatment plants (WWTPs) cannot abate these complex molecules that, often unmodified, re-enter the aquatic compartment. For these reasons, recent European directives encourage developing new strategies to reduce pharmaceuticals from waters, especially antibiotics [33]. These pose a severe threat because they can generate antibiotic-resistant bacteria (ABR), a phenomenon not easily avoided by reducing the only antibiotic consumption [34]. Adsorption is considered an alternative, but consolidated technique for CEC water remediation [35]. Despite many advantages, such as low cost, high efficiency and easy conventional WWTPs integration, many research efforts are being done to develop and test new adsorbent materials for removing such persistent molecules from waters. Adsorbent materials characterized by high surface area, high stability in aqueous media, low toxicity, such as MIL100(Fe), are promising and also complied with the principles of green analytical chemistry [36]. On the other hand, they are not easy to separate from the aqueous medium after the adsorption treatment. In this concern, many works have been carried on to prepare magnetic metal-organic frameworks (MMOFs) by combining the outstanding adsorption capacity of MOFs with the magnetic features of magnetic nanoparticles, such as magnetite, Fe_3_O_4_. 

The synthesis routes generally reported in the literature to prepare MMOFs are embedding [37], mixing [38], encapsulation [39], and layer-by-layer [40] techniques. Some limiting factors are envisaged in the first three approaches: (i) the magnetic nanoparticles may occupy MOFs pores, reducing their adsorption efficiency; (ii) they often tend to polymerize together, decreasing the binding efficiency of the composite; (iii) there is limited control on particle size and morphology. In this light, the layer-by-layer approach seems to be more suitable. In a typical synthesis, the magnetic nanoparticles are prepared, carboxyl-functionalized, and covered by MOFs grown shell-by-shell repeating the covering procedure several times (typically 20 times). By following this synthetic route, homogeneous spherical nanoparticles are obtained. On the other hand, this approach, recently reported [41], is undoubtedly time and solvent consuming. 

In the present study, we considered it worthwhile to synthesize Fe_3_O_4_@MIL-100(Fe) by a modified and not yet proposed layer-by-layer approach, specifically designed to reduce the number of the covering steps, maintaining the nanoparticle dimensions and shape/morphology. The synthesized material was characterized by FT-IR, XRPD and SEM analyses; a model was proposed to evaluate the Fe_3_O_4_ and MIL-100(Fe) percentages in the Fe_3_O_4_@MIL-100(Fe) samples by thermogravimetric data.

In comparison with MIL-100(Fe), Fe_3_O_4_@MIL-100(Fe) was tested to remove one of the most used fluoroquinolone antibiotics, Ofloxacin (OFL), under relevant environmental conditions, *viz.* tap and river water, micrograms per liter concentration, low adsorbent loading, following the recent adsorption testing guidelines [36]. Equilibrium and kinetic aspects were also investigated to elucidate the antibiotic adsorption process.

## 2. Materials and Methods

### 2.1. Materials

All the chemicals employed were reagent grade or higher in quality. Fe(NO_3_)_3_·6H_2_O, FeCl_3_·6H_2_O, ethylene glycol (C_2_H_6_O_2_), sodium acetate (CH_3_COONa), trisodium citrate (Na_3_C_6_H_5_O_7_), trimesic acid (H_3_BTC), methanol (MeOH), ethanol (EtOH), OFL and ultrapure hydrochloric acid (HCl, 30% *w*/*w*) were purchased from Merck (Milano, Italy). High-performance liquid chromatography (HPLC) gradient–grade acetonitrile (ACN) was purchased by VWR International (Milano, Italy), H_3_PO_4_ (85% *w*/*w*), MeOH, and water for liquid chromatography/mass spectrometry (LC/MS) by Carlo Erba Reagents (Cornaredo, Milano, Italy). Aqueous OFL solution (293 mg L^−1^) was prepared in tap water and stored in the dark at 4 °C before use.

### 2.2. Synthesis

#### 2.2.1. MIL-100(Fe)

The synthesis of MIL-100(Fe) was performed under HF-free conditions by following the procedure reported by Zhang et al. [42]. Unlike synthetic routes reported in the literature, such as solvothermal and microwave-assisted solvothermal, this synthetic approach exploits the reflux method that does not involve high energy-consuming steps because of atmospheric pressure and low temperature (<100 °C). Fe(NO_3_)_3_·6H_2_O and H_3_BTC in the 10:9 mole ratio were dissolved in 6 mL of tri-distilled water. The solution was purred in a three-neck round bottom flask and magnetically stirred at 95 °C for 15 h. An orange-brownish solid product formed. The suspension was cooled down to room temperature and centrifuged at 6000 rpm for 15 min. The obtained powder was purified by applying several washing processes. The solid was dispersed in 120 mL of hot tri-distilled water (60 °C), filtered, stirred in 20 mL EtOH for 30 min, filtered again, and washed in hot EtOH (50 °C). The final product was dried in an oven at 90 °C overnight. The obtained sample is named MIL-100.

#### 2.2.2. Fe_3_O_4_@MIL-100(Fe)

Fe_3_O_4_@MIL-100(Fe) particles were synthesized by following the approach reported by Yu [41], with some changes to obtain the final product more quickly. Yu research group pointed at preparing superparamagnetic Fe_3_O_4_ supraparticles@MIL-100(Fe) core-shell nanostructures by a feasible step-by-step procedure starting with the synthesis of carboxylate-functionalized Fe_3_O_4_. The carboxylate groups act as Fe_3_O_4_ stabilizers and grabbing agents favourable to the MIL-100 growth on the magnetite surface. The carboxylate-functionalized Fe_3_O_4_ cores were prepared as proposed by Xuan [43], and the MIL-100(Fe) shell was obtained by applying for twenty cycles the procedure used to form the first MIL-100(Fe) shell. The MIL-100(Fe) deposition on the Fe_3_O_4_ cores is undoubtedly a straightforward procedure but time and solvent consuming. In our procedure, the carboxylate-functionalized Fe_3_O_4_ cores were synthesized using sodium citrate as chelating agent, and the MIL-100(Fe) shell was carried on for five cycles, then the MIL-100(Fe) growth was completed by reflux (Fe_3_O_4_@MIL-100_R sample) or hydrothermal (Fe_3_O_4_@MIL-100_H sample) routes. Two samples synthesized by growing MIL-100(Fe) for 5 and 20 cycles, as in [41] were also prepared, for comparison (Fe_3_O_4_@MIL-100_5 and Fe_3_O_4_@MIL-100_20 samples, respectively).

Table 1 summarizes the synthesis procedures used to grow the MIL-100(Fe) on the Fe_3_O_4_.

Hereafter the synthesis procedures are reported in detail.

(A) Fe_3_O_4_ sample

The carboxylate-functionalized Fe_3_O_4_ nanoparticles were prepared by adopting the solvothermal route [43]. 3.46 g FeCl_3_·6H_2_O and 2.66 g sodium acetate were dissolved in 70 mL ethylene glycol under vigorous stirring, and the obtained yellow solution was transferred into a Teflon–lined stainless-steel autoclave, sealed and treated for 8 h at 200 °C. The magnetic product was separated using a magnet, washed in MeOH and tri-distilled water, and dried for 6 h at 60 °C under vacuum. 0.1 g of the product and 0.1 g of trisodium citrate were then dispersed in 80 mL of distilled water, sonicated at room temperature for 5 h, and the obtained carboxylate-functionalized Fe_3_O_4_ was magnetically separated, washed several times, and vacuum-dried at 60 °C for 12 h. The obtained sample was named Fe_3_O_4_. 

(B) Fe_3_O_4_@MIL-100(Fe): layer by layer route

The Fe_3_O_4_ synthesized in (A) was then treated with FeCl_3_·6H_2_O and H_3_BTC to obtain a first MIL-100(Fe) shell following the procedure reported in [41]. In a typical procedure, 0.1 g of carboxylate-functionalized Fe_3_O_4_ and 0.027 g of FeCl_3_·6H_2_O were mixed in 5 mL EtOH at room temperature for 15 min, and the final solid was magnetically separated and washed in EtOH; then the product was added to an aqueous solution of H_3_BTC (0.021 g) and stirred at 70 °C for 30 min. The final product was washed in EtOH and magnetically separated. This first-shell procedure was repeated for 5 cycles to obtain the sample named Fe_3_O_4_@MIL-100_5, and for 20 cycles to synthesize the sample named Fe_3_O_4_@MIL-100_20, reproducing that prepared by the Yu group [41]. 

(C) Fe_3_O_4_@MIL-100(Fe): reflux route

0.1 g of the Fe_3_O_4_@MIL-100_5 nanoparticles were added to the Fe(NO_3_)_3_·6H_2_O and H_3_BTC in the 10:9 mole ratio dissolved in 6 mL of tri-distilled water, and the procedure reported for the MIL-100 synthesis (Section 2.2.1) was applied.

(D) Fe_3_O_4_@MIL-100(Fe): hydrothermal route

The Fe_3_O_4_@MIL-100_5 nanoparticles were dispersed in 50 mL EtOH, and 0.405 g FeCl_3_·6H_2_O and 0.315 g trimesic acid were added. The reagents amount was properly chosen to equalize that used by Yu [41] for 15 deposition steps of the MIL-100 on the magnetic Fe_3_O_4_. The dispersion was transferred into a Teflon-lined stainless-steel autoclave, sealed, and treated at 70 °C for 24 h. The obtained product was magnetically separated and dried.

### 2.3. Characterization Techniques

X-ray powder diffraction (XRPD) measurements were performed using a Bruker D5005 diffractometer (Bruker, Karlsruhe, Germany) with the CuKα radiation, graphite monochromator, and scintillation detector. The patterns were collected in the 3–35°, 21–60° and 3–60° two-theta angular range for the MIL-100(Fe), Fe_3_O_4_ and Fe_3_O_4_@MIL-100(Fe) samples, respectively, step size of 0.03° and counting time of 4 s/step. A silicon low-background sample holder was used. 

FT-IR spectra were obtained with a Nicolet FT-IR iS10 Spectrometer (Nicolet, Madison, WI, USA) equipped with ATR (attenuated total reflectance) sampling accessory (Smart iTR with ZnSe plate) by co-adding 32 scans in the 4000–500 cm^−1^ range at 4 cm^−1^ resolution. 

Thermogravimetric measurements were performed by a TGA Q5000 IR apparatus interfaced with a TA 5000 data station (TA Instruments, Newcastle, DE, USA). The samples (about 3.5–4 mg) were scanned at 10 K min^−1^ under nitrogen flow (45 mL∙min^−1^). Each measurement was repeated at least three times.

Calorimetric measurements were performed by a SDT Q600 apparatus interfaced with a TA 5000 data station (TA Instruments, Newcastle, DE, USA). The samples were scanned at 10 K min^−1^ under nitrogen flow (100 mL min^−1^). The measurements were carried out in open standard alumina pans.

SEM measurements were performed using a Zeiss EVO MA10 (Carl Zeiss, Oberkochen, Germany) Microscope. The SEM images were collected on gold-sputtered samples. HR-SEM images were taken from a FEG-SEM Tescan Mira3 XMU. Samples were mounted onto aluminum stubs using double sided carbon adhesive tape and were then made electrically conductive by coating in vacuum with a thin layer of Pt. Observations were made at 25 kV with an In-Beam SE detector at a working distance of 3 mm.

The surface area of the materials was determined by N_2_ adsorption using BET method in a Sorptomatic 1990 porosimeter (Thermo Electron).

### 2.4. Analytical Measurements

HPLC-UV Shimadzu (Shimadzu Corporation, Milano, Italy) system consisting of an LC-20AT solvent delivery module equipped with a DGU-20A3 degasser and interfaced with SPD-20A UV detector was used for the adsorption equilibrium experiments at mg L^−1^ concentration. The wavelength selected for analysis was 280 nm corresponding to the maximum OFL adsorption. Each sample was filtered (0.22 µm) and injected (20 µL) into a 250 × 4.6 mm, 5 µm KromaPhase 100 C18 (Scharlab, Riozzo di Cerro al Lambro, Milano, Italy) coupled with a similar guard-column. Isocratic elution was carried out by using a mobile phase 25 mM H_3_PO_4_–ACN (85:15). The flow rate was 1.0 mL min^−1^.

Calibration with four standards at concentrations between 1 and 10 mg L^−1^ yielded optimal linearity (R^2^ > 0.9996). The quantification limit was 0.8 mg L^−1^.

For the kinetic experiments and OFL removal at µg L^−1^, HPLC system consisting of a pump Series 200 (Perkin Elmer, Milano, Italy) equipped with a vacuum degasser and a programmable fluorescence detector (FD) was used. The fluorescence excitation/emission wavelengths selected were 280/450 nm. Fifty µL of each sample were filtered (0.22 µm nylon syringe filter) and injected into a 250 × 4.6 mm, 5 µm Ascentis RPAmide (Supelco-Merck Life Science, Milano, Italy) coupled with a similar guard-column. The mobile phase was 25 mM H_3_PO_4_–ACN (85:15), the flow rate was 1 mL min^−1^.

Calibration with four standards in the range 1–10 µg L^−1^ yielded optimal linearity (R^2^ > 0.9996). The quantification limit was 0.9 µg L^−1^.

### 2.5. Adsorption Experiments

#### 2.5.1. Adsorption Equilibrium Study

OFL adsorption on the MIL-100(Fe) and Fe_3_O_4_@MIL-100(Fe) was studied using a consolidated batch equilibration method [44]. Briefly, a fixed amount (10 mg) of each MOF was suspended in 20 mL of tap water spiked with OFL in the range 10–293 mg L^−1^. Each flask, wrapped with aluminium foils to avoid fluoroquinolone (FQ) light-induced decomposition, was gently shaken in an orbital shaker for 24 h at room temperature. After equilibration, the supernatant from MIL-100(Fe) was separated by filtration on 0.22 µm nylon syringe filter and the one from Fe_3_O_4_@MIL-100(Fe) magnetically, before HPLC–UV analysis. 

The adsorbed OFL amount (*q_e_*, mg g^−1^) was calculated as the difference from the total drug amount initially present and that still in solution after equilibration (Equation (1)):

(1) $$q_{e}=\frac{\left(C_{0}-C_{e}\right)\times V}{M}$$

where *C*_0_ is the initial OFL concentration (mg L^−1^), *C_e_* the OFL concentration in solution at equilibrium (mg L^−1^), *V* the volume of the solution (L), and *M* the amount of the adsorbent (g).

All experiments were performed in duplicate. 

Control samples not containing MOF were analyzed, and no change in OFL concentration was detected. 

The isotherm parameters were calculated by dedicated software (OriginPro, Version 2019b. OriginLab Corporation, Northampton, MA, USA).

#### 2.5.2. Kinetic Study

20 mg of MIL-100(Fe) and Fe_3_O_4_@MIL-100(Fe) were separately suspended in 40 mL of tap water at an initial OFL concentration of 20 mg L^−1^. Unlike the previous paper [44], the suspensions were mandatorily stirred with a glass stirring rod throughout the experiment. Aliquots (100 µL) were withdrawn at various time intervals in the range of 0–60 min, diluted to 5–10 mL tap water, and filtered (0.22 µm nylon syringe filter) before the HPLC-FD analysis for the OFL determination at time *t* (*C_t_*).

The following equation calculated the adsorbed OFL amount at time t (*q_t_*, mg g^−1^):

(2) $$q_{t}=\frac{\left(C_{0}-C_{t}\right)\times V}{M}$$

where *C*_0_ is the initial OFL concentration (mg L^−1^), *C_t_* the OFL concentration in solution at time *t* (mg L^−1^), *V* the volume of the solution (L), and *M* the amount of the adsorbent (g).

All experiments were performed in duplicate. 

Control samples not containing MIL-100(Fe) and Fe_3_O_4_@MIL-100(Fe) were analyzed, and no change in OFL concentration was detected. 

The kinetic parameters were calculated by dedicated software (OriginPro, Version 2019b. OriginLab Corporation, Northampton, MA, USA).

## 3. Results and Discussion

In the present work, the Fe_3_O_4_, MIL-100(Fe) and Fe_3_O_4_@MIL-100(Fe) samples were characterized as concerns the structure, morphology and composition. The OFL antibiotic removal efficiency of the well-known iron-based Metal-Organic Framework, MIL-100(Fe), was tested under actual conditions and compared with the magnetic one, synthesized by the combined layer-by-layer/hydrothermal route (Fe_3_O_4_@MIL-100_H sample).

### 3.1. Fe_3_O_4_, MIL-100(Fe) and Fe_3_O_4_@MIL-100(Fe) Characterization 

**Figure 1:**
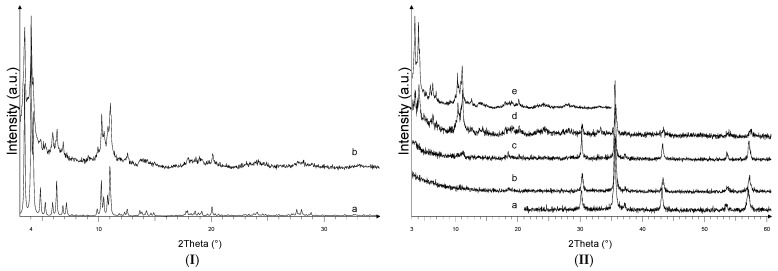
XRPD patterns of (**I**) MIL-100: simulated (pattern a) and synthesized (pattern b), and (**II**) Fe_3_O_4_ (pattern a), Fe_3_O_4_@MIL-100_5 (pattern b), Fe_3_O_4_@MIL-100_20 (pattern c), Fe_3_O_4_@MIL-100_H (pattern d), MIL-100 (pattern e).

In Figure 1I, the X-ray powder diffraction pattern of the MIL-100 sample is shown and compared to the simulated one, based on the crystal structure reported in the literature [16] and deposited in the Cambridge Crystallographic Data Centre (CCDC 640536). The diffraction patterns display comparable peak positions and intensities, suggesting the MIL-100 sample is crystalline and successfully synthesized. 

Figure 1II displays the diffraction pattern of the Fe_3_O_4_, and Fe_3_O_4_@MIL-100(Fe) samples. The diffraction data of the MIL-100 are also shown for comparison. As concerns the Fe_3_O_4_@MIL-100_R sample, synthesized by the reflux technique approach, a bi-phasic mixture is obtained. The sample contains black magnetic agglomerates and orange-brownish non-magnetic ones, thus suggesting the MIL-100(Fe) phase (orange brownish) is not grown on the magnetite particles (black) but crystallizes as a separate phase. This hypothesis is confirmed by the X-ray powder diffraction analysis of the black fraction and of the orange-brownish one (data not shown). These evidences suggest the reflux approach is not suitable to successfully grow the MIL-100(Fe) on the Fe_3_O_4_ nanoparticles, and the Fe_3_O_4_@MIL-100_R sample was not further considered. In the case of the Fe_3_O_4_@MIL-100_5, Fe_3_O_4_@MIL-100_20, and Fe_3_O_4_@MIL-100_H samples, the powders could be fully recovered by a magnet, thus suggesting the MIL-100(Fe) phase is linked to the magnetite particles. The diffraction peaks observed in Figure 1II for the Fe_3_O_4_ sample reasonably agree with those reported for the magnetite structure in the JCPDS database (PDF# 088-0315), in which the peaks at about 30.2°, 35.5°, 37.2°, 43.2°, 53.6°, and 57.1° are attributed to the (220), (311), (222), (400), (422) and (511) planes of the Fe_3_O_4_. 

The Fe_3_O_4_@MIL-100(Fe) samples display the peaks of the Fe_3_O_4_ magnetite compound and those pertinent to the MIL-100(Fe) phase, thus confirming the desired crystalline phases were obtained in different amounts, depending on the sample. The MIL-100(Fe) reflections are clearly detected in the Fe_3_O_4_@MIL-100_H sample. Instead, they are weaker and broader in the Fe_3_O_4_@MIL-100_20 sample. This suggests the step-by-step procedure up to 5 cycles followed by the hydrothermal route is suitable to obtain Fe_3_O_4_@MIL-100(Fe) composite with higher crystallinity degree and amount of the MIL-100(Fe). As concerns the Fe_3_O_4_@MIL-100_5 sample, the peaks pertinent to the MIL-100(Fe) are poorly detected, in agreement with the expected smaller quantity of MIL-100(Fe) in the composite treated only for 5 cycles. The X-ray powder diffraction results suggest the step-by-step route combined with the hydrothermal one represents a feasible and quick strategy to synthesize magnetite particles coated with MIL-100(Fe).

**Figure 2:**
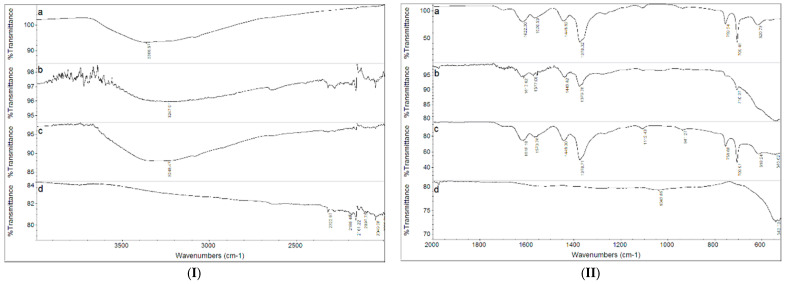
FT-IR spectra of the samples MIL-100 (spectrum a), Fe_3_O_4_@MIL-100_20 (spectrum b), Fe_3_O_4_@MIL-100_H (spectrum c), and Fe_3_O_4_ (spectrum d) in (**I**) 4000–2000 cm^−1^ and (**II**) 2000–500 cm^−1^ wavenumber range.

The FT-IR spectra of the MIL-100, Fe_3_O_4_@MIL-100_20, Fe_3_O_4_@MIL-100_H and Fe_3_O_4_ samples are shown in Figure 2. The FT-IR spectrum of the MIL-100 is comparable to that reported in the literature [18,45] and puts into evidence the presence of the organic linker and the –OH groups related to the coordination water molecules. The broad band centred at about 3400 cm^−1^ is related to the –OH stretching of the carboxylic groups, while the weak peak at 3077 cm^−1^ and the stronger ones at 760 and 709 cm^−1^ are attributed to the C–H stretching of the benzenic ring. The bands at 1703 and 1622 cm^−1^ are assigned to the C=O stretching and the intense band at 1378 cm^−1^ to the C–O bonds vibration. The –OH bond vibration explains the band at 1449 cm^−1^. In the Fe_3_O_4_@MIL-100_20 and Fe_3_O_4_@MIL-100_H samples, the relevant bands of the MIL-100 are detected, confirming the presence of this phase in the samples; the bands are more evident in the Fe_3_O_4_@MIL-100_H sample. 

**Figure 3:**
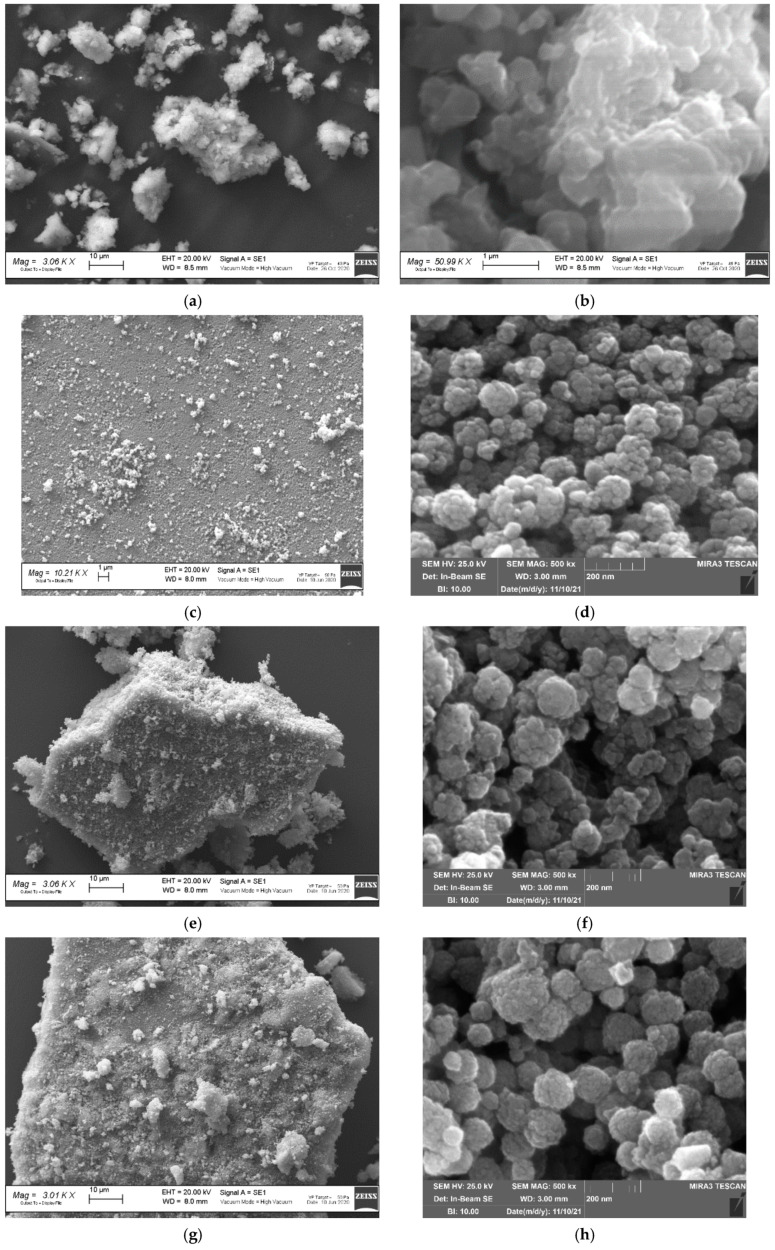

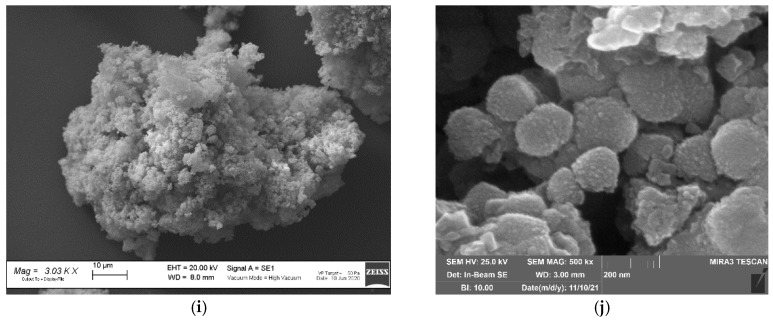
SEM and HR-SEM images of (**a**,**b**) MIL-100, (**c**,**d**) Fe_3_O_4_, (**e**,**f**) Fe_3_O_4_@MIL-100_5, (**g**,**h**) Fe_3_O_4_@MIL-100_20, (**i**,**j**) Fe_3_O_4_@MIL-100_H samples.

Figure 3 shows the SEM and HR-SEM micrographs of the MIL-100, Fe_3_O_4,_ and Fe_3_O_4_@MIL-100(Fe) samples. The MIL-100 displays large agglomerates composed of micro-sized irregular particles with smooth surfaces. The Fe_3_O_4_ sample displays rounded-shaped agglomerates of about 100 nm, in which nanometric sub-units of 20–50 nm diameter are observed. The rounded morphology of the aggregates is preserved after the MIL-100(Fe) shell growth, independently of the synthesis method (layer-by-layer alone or combined layer-by-layer/hydrothermal). As concerns the agglomerates size, it slightly increases in the Fe_3_O_4_@MIL-100_20 sample and reaches 200–400 nm values in the Fe_3_O_4_@MIL-100_H one. As for aggregates surface, in the Fe_3_O_4_@MIL-100_20 sample, the sub-units are less defined due to the covering layer of MOF. This effect is magnified in the Fe_3_O_4_@MIL-100_H sample. In the latter, the MOF covering the aggregate surface is nanometric with a particles size of about 20 nm. The Fe_3_O_4_@MIL-100_H morphology and the very small particle size of the MIL-100(Fe) layer suggest the sample could display a high surface area. The N_2_ adsorption isotherms of the Fe_3_O_4_@MIL-100_H and MIL-100 samples are shown in Figure S1. From BET measurements, we obtained a surface area of 3546 m^2^∙g^−1^ for Fe_3_O_4_@MIL-100_H and 1199 m^2^∙g^−1^ for MIL-100. 

The results obtained by SEM and HR-SEM investigation, consistent with the X-ray powder diffraction and FT-IR results, highlight the higher amount of the MIL-100(Fe) grown on the Fe_3_O_4_ spherical cores by hydrothermal synthesis, despite the same reagents amount was used in the two approaches. The mixed layer-by-layer/hydrothermal route successfully synthesizes spherical Fe_3_O_4_@MIL-100(Fe) nanoparticles richer in MIL-100(Fe) amount than the 20 shells one, with a simplified, not time and solvent consuming procedure.

The thermogravimetric technique was revealed to be a powerful tool to evaluate the MIL-100(Fe) amount in the Fe_3_O_4_@MIL-100(Fe) composites. This is useful when comparing the absorption efficiency of the MIL-100 and the Fe_3_O_4_@MIL-100_H samples toward OFL.

**Figure 4:**
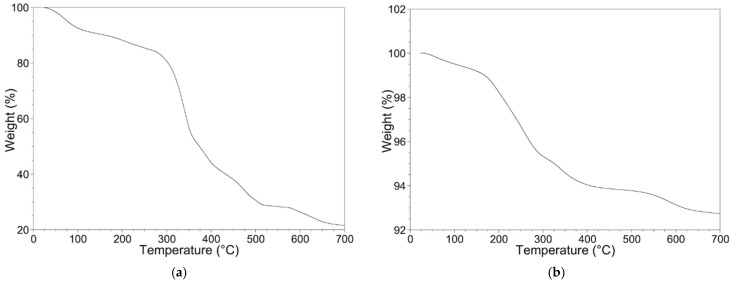

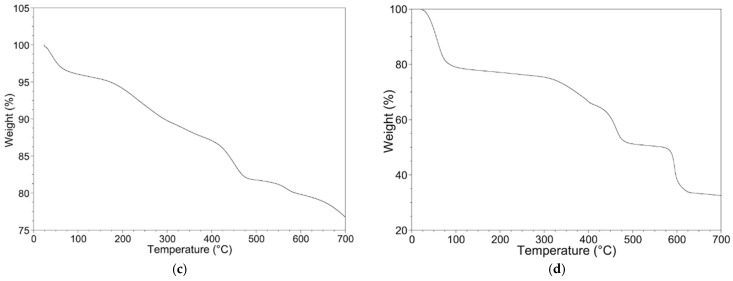
TGA curves of (**a**) MIL-100 (**b**) Fe_3_O_4_, (**c**) Fe_3_O_4_@MIL-100_20, (**d**) Fe_3_O_4_@MIL-100_H samples.

In fair agreement with the literature [42,46], the TGA curve of the MIL-100 sample (Figure 4a) shows several mass losses that can be easily explained: (1) desorption of the free water molecules present in the pores in the 25–110 °C temperature range; (2) removal of water molecules coordinated to Iron trimers in the 110–260 °C temperature range; (3) H_3_BTC combustion in the 260–530 °C temperature range; (4) decomposition of MIL-100 with the formation of FeO after 600 °C.

The TGA curve of the Fe_3_O_4_ sample (Figure 4b) shows a slight mass loss (−0.5%) in the 25–100 °C temperature range likely due to the removal of water molecules adsorbed on the nanoparticles surface, and a multi-step mass loss in the 100–700 °C range due to the Fe_3_O_4_ reduction in N_2_ flow.

The TGA curves of the Fe_3_O_4_@MIL-100_20 and Fe_3_O_4_@MIL-100_H samples are shown in Figure 4c,d, respectively. Both samples display mass losses in several steps.

The DSC curves of the Fe_3_O_4_, Fe_3_O_4_@MIL-100_20, Fe_3_O_4_@MIL-100_H, and MIL-100 samples are shown in Figure S2. The temperatures of the DSC thermal events and TGA mass losses are in fair agreement. 

To determine the MIL-100(Fe) amount in the Fe_3_O_4_@MIL-100(Fe) samples, we developed a model based on the following point: the thermal treatment up to 700 °C in N_2_ flux of both Fe_3_O_4_ and MIL-100 forms FeO, and its amount corresponds to the mass percentage measured at the end of the TGA scan.

The Fe_3_O_4_ sample during heating in N_2_ flow is reduced to FeO according to the reaction (Equation (3)):

Fe_3_O_4_ → 3FeO + ½ O_2_ (3)

The mass percentage of the FeO calculated (*m*%c) on the basis of this reaction (3) is 93.1%, in fair agreement with the experimental value (*m*%s) of 92.8%. The experimental value, however, must be corrected considering that the sample contains a small amount of adsorbed water which is released by the sample in the 25–110 °C temperature range. The value corrected by subtracting the mass loss observed in the 25–110 °C range and re-scaling the obtained mass to 100% (*m*%*) is 93.2% (Table 2), in very good agreement with the calculated one. 

For the MIL-100 sample, we hypothesize a decomposition to iron oxides that subsequently reduce to FeO under N_2_ flow. MIL-100 and the product FeO are in the 1:3 molar ratio. The mass percentage calculated on the basis of these processes is 23.6% (Table 2) and well agrees to the experimental one, corrected for the mass loss due to adsorbed molecules released in the 25–110 °C temperature range (*m*%*).

Thus, the experimental results support the hypothesis of FeO formation for thermal treatment at 700 °C under N_2_ flux of both MIL-100 and Fe_3_O_4_. This evidence can be used to evaluate the Fe_3_O_4_ and MIL-100 masses percentage in the Fe_3_O_4_@MIL-100_20 and Fe_3_O_4_@MIL-100_H samples. A simple equation can be formulated, known the molar masses and molar ratios of MIL-100, Fe_3_O_4_, and FeO:

(4) $$m{\%}^{*}=3\cdot PM_{\left(FeO\right)}\left[\frac{x}{PM_{\left(MIL100\right)}}+\frac{100-x}{PM_{\left(Fe_{3}O_{4}\right)}}\right],$$

where *x* is the mass percentage of MIL-100 in the sample, and *m*%* the mass values obtained at 700 °C by TGA curves, corrected by subtracting the mass loss observed in the 25–100 °C range and re-scaling the obtained mass to 100%. The results are reported in Table 2.

The obtained results agree with the X-ray powder diffraction ones, showing a higher amount of MIL-100 coated on the Fe_3_O_4_ cores by using the hydrothermal approach with respect to the step-by-step one. The same amount of reagents was used in both procedures, thus suggesting a higher reaction yield in the hydrothermal route to carry on the MIL-100 growth on the Fe_3_O_4_ cores. The MIL-100 percentage calculated by TGA analysis is useful to evaluate the adsorption performance of MIL-100 and Fe_3_O_4_@MIL-100_H towards OFL antibiotic. 

### 3.2. Preliminary Adsorption Experiments

Preliminary adsorption experiments were carried out in tap water due to its remarkable similarity to environmental waters, in terms of pH, ionic strength, and dissolved organic matter, and its invariant composition over time (see Appendix A) that allowed comparing MIL-100(Fe) and Fe_3_O_4_@MIL-100(Fe) adsorption performances easily. Moreover, the above-mentioned parameters may significantly affect the adsorption process [32,44,47,48]. As concerns the tests on the magnetic MOF, the synthesized Fe_3_O_4_@MIL-100_H sample was chosen, due to its high MIL-100(Fe) content (72.7 wt%), good degree of crystallinity and morphology compared to Fe_3_O_4_@MIL-100_5 and Fe_3_O_4_@MIL-100_20 ones. 

Initially, 10 mg of MIL-100 and Fe_3_O_4_@MIL-100_H were separately suspended in 20 mL of tap water not containing the drug and shaken (90 rpm) for 24 h at room temperature. Then the supernatants were separated for the pH measurement. MIL-100 blank sample had a pH value of 4.2, while the measured pH in Fe_3_O_4_@MIL-100_H blank sample was around 7. Consequently, MIL-100 was rinsed with tap water and EtOH to eliminate any residuals from the material before use. After washing and air drying, 10 mg of MIL-100 was re-suspended in 10 mL of tap water not containing the drug and treated as above, obtaining a pH value of around 7. On the contrary, no additional treatment was necessary for Fe_3_O_4_@MIL-100_H. 

### 3.3. Isotherm and Kinetic Studies

#### 3.3.1. Isotherm Study

The maximum adsorption uptake and the adsorption process can be evaluated using models that relate the amount of the adsorbed molecule (*q_e_*) to the molecule concentration (*C_e_*) in the solution at equilibrium. In this paper, the most frequently used models, i.e., Freundlich and Langmuir, and Brunauer-Emmett-Teller (BET) models, were applied to fit the experimental profile and to quantitatively describe the maximum uptake of OFL. 

The Freundlich model describes non-ideal adsorption on the heterogeneous surface, and it is expressed by Equation (5):

(5) $$q_{e}=K_{F}C_{e}^{1/n}$$

where *K_F_* is the empirical constant indicative of adsorption capacity, and *n* is the empirical parameter representing the heterogeneity of site energies.

The Langmuir model (Equation (6)) assumes that the adsorption process occurs in a mono-layer that covers the surface of the adsorbent:

(6) $$q_{e}=\frac{q_{m}K_{L}C_{e}}{1+K_{L}C_{e}}$$

where *K_L_* is the Langmuir constant and *q_m_* the mono-layer saturation capacity.

BET isotherm for liquid-phase adsorption describes [44] multi-layer adsorption:

(7) $$q_{e}=q_{m}\frac{K_{s}C_{eq}}{\left(1-K_{L}\right)\left(1-K_{L}C_{eq}+K_{s}C_{eq}\right)}$$

where *K_L_* is the adsorption equilibrium constant of potential upper layers and *K_S_* equilibrium constant for the first layer.

**Figure 5:**
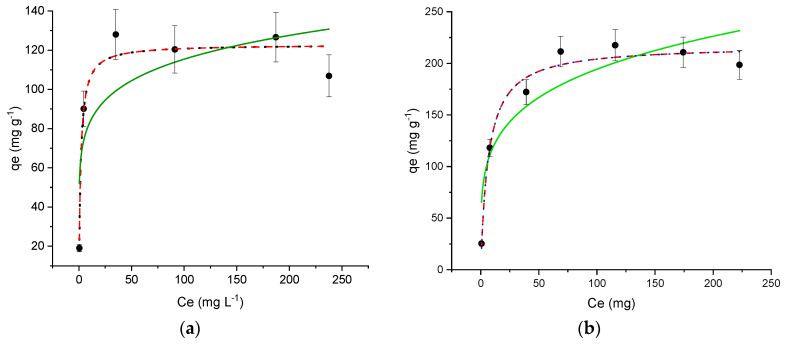
Adsorption profiles Langmuir (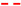), Freundlich (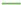), BET (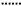) for Ofloxacin (OFL) on (**a**) MIL-100 and (**b**) Fe_3_O_4_@MIL-100_H. (Experimental conditions: MOF 10 mg, 20 mL OFL solution from 10 to 293 mg L^−1^; RDS ≤ 10%).

Figure 5a,b show the OFL experimental adsorption profiles on MIL-100 and Fe_3_O_4_@MIL-100_H. A similar trend was observed, with a maximum uptake of 123 ± 5 mg g^−1^ for MIL-100 and 218 ± 7 mg g^−1^ for Fe_3_O_4_@MIL-100_H. 

The isotherm parameters, calculated by dedicated software, are listed in Table 3.

The good Langmuir correlation coefficient, R^2^, proves that mono-layer drug adsorption occurred on both MOF and the experimental *q_m_* values are not significantly different from those calculated from the model. *K_L_, K_s_* and *q_m_* values from BET model confirmed this behavior.

Interestingly, a remarkably higher adsorption uptake was observed for Fe_3_O_4_@MIL-100_H than MIL-100. This trend is unexpected due to the MIL-100 amount, equal to 72.7% (see Table 2), present in the magnetic composite. In addition, a satisfactory *q_m_* was obtained despite the presence of matrix constituents, differently from data reported by Moradi et al. [49]. They investigated FQs adsorption on Fe_3_O_4_@MIL-100(Fe) and Fe_3_O_4_@MOF-235(Fe) under ideal conditions (ultrapure water), obtaining higher adsorption capacity values that drastically decreased (up to 5 times) in the presence of salts, especially divalent cations, such as Ca^2+^. Undoubtedly, both the morphology and the particle dimensions of Fe_3_O_4_@MIL-100_H synthesized in the present work favoured the adsorption process, as well as the high surface area of 3546 m^2^ g^−1^.

Although both iron-based materials have an excellent affinity towards OFL, Fe_3_O_4_@MIL-100_H is undoubtedly more attractive due to its higher adsorption capacity—comparable to that of the most traditional activated carbon [50]—and, significantly, its magnetic properties that allow it to be easily separated from the medium using an external magnetic field. 

#### 3.3.2. Kinetic Study

Among various adsorption kinetic mechanistic models, pseudo-first-order (Equation (8)) and pseudo-second-order (Equation (9)) equations are used to describe OFL uptake on the two investigated MOFs:

(8) $$q_{t}=q_{e}\left(1-e^{-k_{1}t}\right),$$

(9) $$q_{t}=\frac{\left(q_{e}^{2}k_{2}t\right)}{\left(1+q_{e}k_{2}t\right)},$$

where *q_t_* and *q_e_* were the OFL amount adsorbed at time *t* and equilibrium, respectively, *k*_1_ was the pseudo-first-order rate constant, *k*_2_ the pseudo-second-order rate constant.

**Figure 6:**
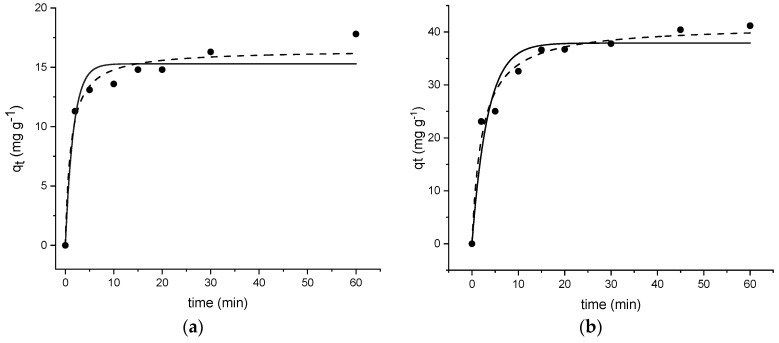
Kinetic profiles (pseudo-first-order (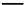), pseudo-second order (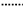) for OFL on (**a**) MIL-100 and (**b**) Fe_3_O_4_@MIL-100_H (Experimental conditions: MOF 20 mg, 40 mL tap water, OFL initial concentration 20 mg L^−1^; RDS ≤ 10%).

As shown in Figure 6a,b, the experimental kinetic profiles obtained under the same experimental conditions are markedly similar. Moreover, the experimental equilibrium uptakes (*q_e exp_*) are in good agreement with the calculated ones.

The kinetic parameters obtained by fitting the experimental data are shown in Table 4.

Based on the correlation coefficients (R^2^) and the good agreement between the experimental adsorption capacities and the calculated ones, we can assume that the pseudo-second-order model is more suitable than the pseudo-first-order one to describe the overall kinetic process, suggesting that chemisorption is the rate-controlling step. OFL adsorption on MIL-100 and Fe_3_O_4_@MIL-100_H would likely result in several interactions, mainly, π-π interactions that occur between delocalized π-electrons in OFL and H_3_BTC in MOFs, and electrostatic interactions between the zwitterionic form of OFL (pK_a1_ = 5.97 carboxylic acid; pK_a2_ = 9.28 piperazinyl ring [51]), dominant in the experimental working conditions (see Appendix A), and the polarized groups on the surface of the adsorbents. Besides, coordination bonds between the open metal sites in MOFs and the carboxylic groups in OFL could not be excluded, and H-bonding interactions between OFL and the hydroxyl group of MOFs [32,47,52,53,54].

#### 3.3.3. Ofloxacin Removal under Relevant Environmental Conditions

MIL-100 and Fe_3_O_4_@MIL-100_H efficiency for OFL removal at a few µg per liter concentrations from freshwaters were investigated.

10 mg of each MOF was suspended in 20 mL of freshwater samples (tap and river) spiked with 10 µg L^−1^ OFL (*C*_0_) and stirred overnight. Then, the supernatant from MIL-100(Fe) was separated by filtration on 0.22 µm nylon syringe filter and that from Fe_3_O_4_@MIL-100(Fe) magnetically before analysis by HPLC-FD to determine the drug content (*C_t_*). 

The removal efficiency (R) was calculated following Equation (10):

(10) $$R\%=\frac{\left(C_{0}-C_{t}\right)}{C_{0}}\times 100$$

where *C*_0_ is the initial OFL concentration and *C_t_* the OFL concentration in solution after a contact time *t*.

The obtained results were reported in Table 5. 

A satisfactory antibiotic removal was obtained in the presence of matrix constituents with both MOFs. Nevertheless, the magnetic behaviour of Fe_3_O_4_@MIL-100_H makes it considerably competitive because the drug removal was quantitative and the adsorbent can be easily recovered. 

## 4. Conclusions

In the present work MIL-100(Fe) and Fe_3_O_4_@MIL-100(Fe) were synthesized and applied as adsorbent materials for the removal of Ofloxacin in environmental conditions, such as tap and river water. The synthesis of the Fe_3_O_4_@MIL-100(Fe) composites, through the combined layer-by-layer (5 shell)/hydrothermal one revealed to be really interesting and advantageous with respect to the layer-by-layer alone (20 shell). Higher yields of MIL-100(Fe) covering the magnetite cores are obtained by using the same reagents amount, with less solvent and time consumption, thanks to the decreased number of covering steps (5 vs. 20). Rounded-shaped nanoparticles with diameters ranging in the 200–400 nm were successfully synthesized, as demonstrated by XRPD and SEM, and a surface area of 3546 m^2^ g^−1^ is obtained, higher than that of the MIL-100(Fe) alone. Moreover, a suitable decomposition-reduction model was developed to explain the mass percentage observed at 700 °C in the TGA curves. This datum was used to evaluate the MIL-100(Fe) amount in the Fe_3_O_4_@MIL-100(Fe) samples. 

The monolayer OFL adsorption on Fe_3_O_4_@MIL-100_H indicated an adsorption capacity of 218 ± 7 mg g^−1^, a value quite different from that obtained with MIL-100 (123 ± 5 mg g^−1^), also in the presence of aqueous matrix constituents; the kinetic process, which occurred within 15 min, was regulated by chemisorption. The efficiency of Fe_3_O_4_@MIL-100_H in the OFL adsorption under environmental conditions (µg per liter drug concentration, environmental waters) was even higher than the MIL-100. Moreover, despite the small amount of magnetite (27.3 wt% Fe_3_O_4_) present in the magnetic composite, Fe_3_O_4_@MIL-100_H was easily magnetically recovered after the treatment and, therefore, suitable for large-scale applications.

## Figures and Tables

**Table 1:** Scheme of the synthesis procedures used to prepare Fe_3_O_4_@MIL-100(Fe).

Synthesis Procedure	Layer by Layer 5 Shell	Layer by Layer 20 Shell	Layer by Layer 5 Shell + Reflux	Layer by Layer 5 Shell + Hydrothermal
Sample name	Fe_3_O_4_@MIL-100_5	Fe_3_O_4_@MIL-100_20	Fe_3_O_4_@MIL-100_R	Fe_3_O_4_@MIL-100_H

**Table 2:** Mass% values observed at 700 °C by TGA curves (*m*%s), corrected for adsorbed water (*m*%*), and calculated on the basis of the decomposition-reduction model (*m*%c). Mass percentage of the Fe_3_O_4_ and MIL-100 ((1 − *x*) and *x* in Equation (4)) evaluated in the Fe_3_O_4_@MIL-100_20 and Fe_3_O_4_@MIL-100_H samples.

Sample	*m*%s	*m*%*	*m*%c	Fe_3_O_4_ Mass%	MIL-100 Mass%
Fe_3_O_4_	92.8	93.2	93.1	-	-
MIL-100	21.7	24.0	23.6	-	-
Fe_3_O_4_@MIL-100_20	76.9	80.2	-	81.5	18.5
Fe_3_O_4_@MIL-100_H	33.6	42.9	-	27.3	72.7

**Table 3:** Isotherm parameters for OFL adsorption onto MIL-100 and Fe_3_O_4_@MIL-100_H.

Adsorption Model	Isotherm Parameters	MIL-100	Fe_3_O_4_@MIL-100_H
	*q_m_* exp (mg g^−1^)	127	199
Freundlich	*K_F_* (mg^(1-n)^ L^n^ g^−1^)	59 ± 18	71 ± 19
	1/n	0.14 ± 0.07	0.22 ± 0.07
	R^2^	0.594	0.824
Langmuir	*K_L_* (L mg^−1^)	0.6 ± 0.2	0.15 ± 0.03
	*q_m_* (mg g^−1^)	123 ± 5	218 ± 7
	R^2^	0.944	0.972
BET	*K_s_* (L mg^−1^)	0.6 ± 0.2	0.15 ± 0.04
	*K_L_* (L mg^−1^)	0	6.7E-18 ± 0
	*q_m_* (mg g^−1^)	123 ± 6	218 ± 7
	R^2^	0.925	0.965

**Table 4:** Kinetic parameters for OFL adsorption onto MIL-100 and Fe_3_O_4_@MIL-100_H.

Kinetic Model	Kinetic Parameter	MIL-100	Fe_3_O_4_@MIL-100_H
	*q_e_* exp (mg g^−1^)	18.3	45.3
Pseudo-first order	*k_1_* (min^−1^)	0.6 ± 0.2	0.30 ± 0.06
	R^2^	0.929	0.928
	*q_e_* (mg g^−1^)	15.3 ± 0.6	38 ± 1
Pseudo-second order	*k_2_* (g mg^−1^ min^−1^)	0.05 ± 0.02	0.015 ± 0.003
	R^2^	0.927	0.974
	*q_e_* (mg g^−1^)	16.5 ± 0.6	41 ± 1

**Table 5:** OFL removal efficiency (R%) in tap and river water samples (Experimental conditions: adsorbent 10 mg, 20 mL water sample, OFL concentration 10 µg L^−1^).

	R%	R%
MOF	Tap Water	River Water
MIL-100	88	80
Fe_3_O_4_@MIL-100_H	95	87

RSD % ≤ 10.

## Data Availability

The data presented in this study are available on request from the corresponding author.

## Supplementary Materials

The following are available online at https://www.mdpi.com/article/10.3390/nano11123275/s1, Figure S1: N_2_ adsorption isotherms; Figure S2: DSC curves.

## Appendix A

**Table A1:** Physico-chemical characterization of tap and river water samples.

Parameters/Ions		Tap Water	River Water
pH		7.7	7.9
Conductivity at 20 °C	µS cm^−1^	278	297
TOC	mg L^−1^	<2	4.9
Cl^−^	mg L^−1^	4.5	3.8
NO_3_^−^	mg L^−1^	0.6	1.5
SO_4_^2−^	mg L^−1^	5.0	12.5
HCO_3_^−^	mg L^−1^	195	200
Ca^2+^	mg L^−1^	38	56
Mg^2+^	mg L^−1^	12	7.0
Na^+^	mg L^−1^	11	5.0
