# Supplementary material for: Combined Layer-by-Layer/Hydrothermal Synthesis of Fe3O4@MIL-100(Fe) for Ofloxacin Adsorption from Environmental Waters
Source: Nanomaterials (Basel). 2021 Dec 2;11(12):3275. doi: 10.3390/nano11123275 (PMC8703555; doi:10.3390/nano11123275)
Supplement: Supplementary file 1 [file nanomaterials-11-03275-s001.zip › nanomaterials-1458092-supplementary.pdf]

nanomaterials

Supplementary Materials

Combined Layer-by-Layer/Hydrothermal Synthesis of Fe_3O_4@MIL-100(Fe) for Ofloxacin Adsorption from Environmental Waters

Michela Sturini [1], Constantin Puscalau [2,3], Giulia Guerra [1,4], Federica Maraschi [1], Giovanna Bruni [2], Francesco Monteforte [2], Antonella Profumo[1] and Doretta Capsoni [2,*]

[1] Department of Chemistry, University of Pavia, 27100 Pavia, Italy; michela.sturini@unipv.it (M.S.); giulia.guerra@itb.cnr.it (G.G.); federica.maraschi@unipv.it (F.M.); antonella.profumo@unipv.it (A.P.)
[2] C.S.G.I. (Consorzio Interuniversitario per lo Sviluppo dei Sistemi a Grande Interfase) & Department of Chemistry, Physical Chemistry Section, University of Pavia, 27100 Pavia, Italy; constantin.puscalau@nottingham.ac.uk (C.P.); giovanna.bruni@unipv.it (G.B.); francesco.monteforte01@universitadipavia.it (F.M.)
[3] The GlaxoSmithKline Neutral Laboratories for Sustainable Chemistry, University of Nottingham, Jubilee Campus, NG7 2TU Nottingham, UK
[4] Istituto di Tecnologie Biomediche, ITB-CNR, 20054 Segrate, Milano, Italy
* Correspondence: doretta.capsoni@unipv.it; Tel.: +39-0382-987213

N_2 adsorption data

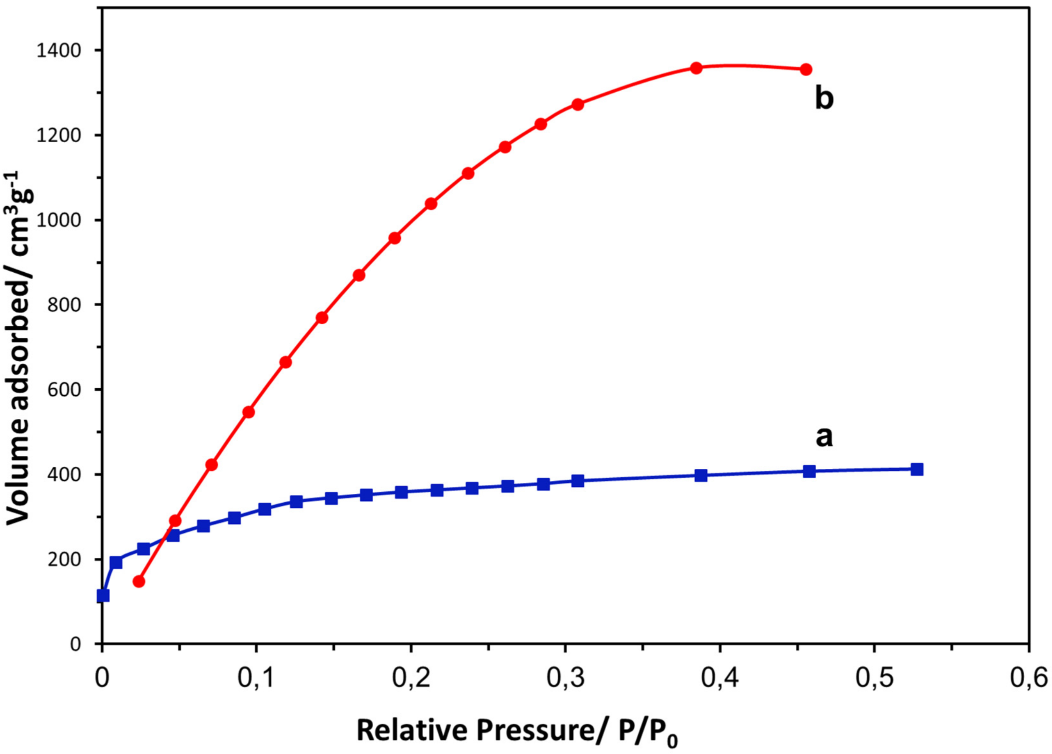

Figure S1. N_2 adsorption isotherms of the **a**) MIL-100 and **b**) Fe_3O_4@MIL-100_H samples.

DSC data

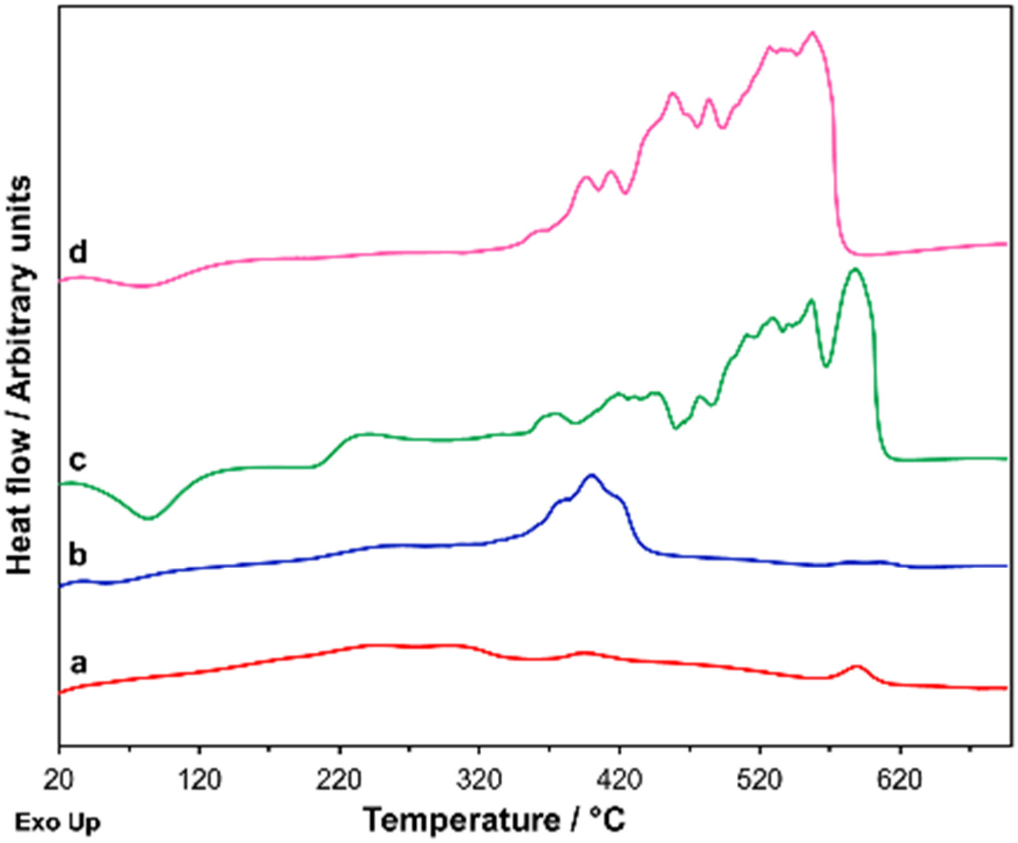

Figure S2. DSC curves of the **a**) Fe_3O_4, **b**) Fe_3O_4@MIL-100_20, **c**) Fe_3O_4@MIL-100_H and **d**) MIL-100 samples.

The DSC curves of the Fe_3O_4, Fe_3O_4@MIL-100_20, Fe_3O_4@MIL-100_H and MIL-100 samples are shown in Figure S2. In the Fe_3O_4 sample (curve a) the temperatures of the DSC thermal events and TGA mass losses are in fair agreement. The peaks are attributed to oxygen loss and Fe_3O_4 reduction to FeO (Figure 4b). The MIL-100 sample (curve d) displays an endothermic peak in the 20-120°C temperature range, related to the desorption of the free water molecules present in the pores (Figure 4a). The very complex signal observed at higher temperatures relates to the H_3BTC combustion and MIL-100 decomposition to form FeO, in fair agreement with the TGA results. The DSC curve of the Fe_3O_4@MIL-100_H sample (curve c) fairly compares to the MIL-100 one, but some peaks in the 150-300°C temperature range possibly related to the presence of Fe_3O_4 in the sample are also observed. We remind the high MIL-100 amount detected in the Fe_3O_4@MIL-100_H sample (72.7 wt%, Table 2). The DSC curve of the Fe_3O_4@MIL-100_20 sample (curve b) fairly compares to the Fe_3O_4 one but some peaks in the 300-450°C temperature range possibly related to the MIL-100 are also observed. We underline that a low MIL-100 amount was detected in the Fe_3O_4@MIL-100_20 sample (18.5 wt%, Table 2).
